# COVID Diaries, State Response to COVID Vaccination Program, December 2020 to September 2021

**DOI:** 10.1038/s41597-026-06975-0

**Published:** 2026-03-20

**Authors:** Avalon S. Moore, Bridget Vitu, Felicia Fraizer-Bisner, Peter J. Williams, Lucy van der Merwe, Abdelrhman Gouda, Dessislava Kirilova, Christopher Pittenger, Helen Pushkarskaya

**Affiliations:** 1 Department of Psychiatry, Yale School of Medicine, New Haven, CT USA; 2 Tobin Center for Economic Policy, Yale University, New Haven, CT USA; 3 Qualitative Data Repository, Maxwell School of Citizenship and Public Affairs, Syracuse University, Syracuse, NY USA; 4 Wu Tsai Institute, Yale University, New Haven, CT USA; 5 Child Study Center, Yale School of Medicine, New Haven, CT USA; 6 Center for Brain and Mind Health, Yale School of Medicine, New Haven, CT USA; 7 Department of Economics, Yale University, New Haven, CT USA

**Keywords:** Government, Health policy

## Abstract

National COVID-19 response plans in the United States recognized that the primary responsibility for addressing domestic health emergencies lay with states and localities, though each state’s pandemic response authority varied. States utilized a range of tools to manage infectious-disease outbreaks, including vaccination rules, incentives, and communication strategies. This database includes online publications from state governors and departments of health across all 50 U.S. states and the District of Columbia. It spans from December 2020, when Phase 1a of the COVID-19 vaccination allocation began, to September 2021, when vaccines were widely available and often mandated. In total, 5,223 unique publications were collected, each classified by type: Flyer, Milestone, Info, and Policy. We also address key considerations for analyzing this data and suggest potential research questions that can be explored with it.

## Background & Summary

On December 12, 2019, a cluster of flu-like symptoms appeared in patients in Wuhan, Hubei Province, China^1^. In the following months, thousands of individuals worldwide contracted what became known as SARS-CoV-2, or COVID-19. By March 2020, the White House declared a national emergency, and the U.S. entered an era of social isolation^1^. It is estimated that by the end of 2020, at least 3 million people had died from the virus^1^. On December 11, 2020, the first COVID-19 vaccine in the U.S. (i.e., Pfizer-BioNTech’s vaccine, closely followed by Moderna and Janssen/Johnson & Johnson) was granted Emergency Use Authorization (EUA) for individuals aged 16 and older, followed by authorization for those aged 12–15 in May 2021^2^.

Through Operation Warp Speed, the U.S. government invested $10 billion to accelerate vaccine development, resulting in EUA for two effective vaccines within a record-breaking 11 months^3^.Although this was a remarkable scientific accomplishment, challenges remained in vaccine acquisition, deployment, and uptake, which were largely handled by states and local governments^4,5^. Each state developed plans to increase capacity, improve data systems, and form partnerships, though approaches varied significantly across regions^6,7^. For instance, vaccine distribution was phased, beginning with Phase 1a for those aged 75 and older, followed by Phase 1b for individuals aged 65–74 or with high-risk medical conditions, Phase 1c for those aged 16–64 with high-risk medical conditions and essential workers, and Phase 2 for those aged 16 and older^1^. By January 26, 2021, all U.S. states had entered Phase 1, though some advanced to Phase 1c while others still remained in Phase 1a^8^.

The success of the U.S. vaccination program depended on effective communication between governments, state health departments, and citizens. However, mistrust and confusion often hindered these efforts^9,10^. Governor Andrew Cuomo of New York, for example, held daily televised briefings to keep the public informed, which served as a highly visible communication model^11^. Other governors and state health departments also worked diligently, though less publicly, to guide their states through the pandemic. Our dataset preserves contemporaneous documents that captured their efforts^12^. Analyses of these documents, in conjunction with other datasets, can help to evaluate how state governments’ communication styles and strategies may be impacted by cultural, socio-economic, or political considerations, and whether their strategies were associated with the efficacy of vaccination programs across states.

**Fig. 1:**
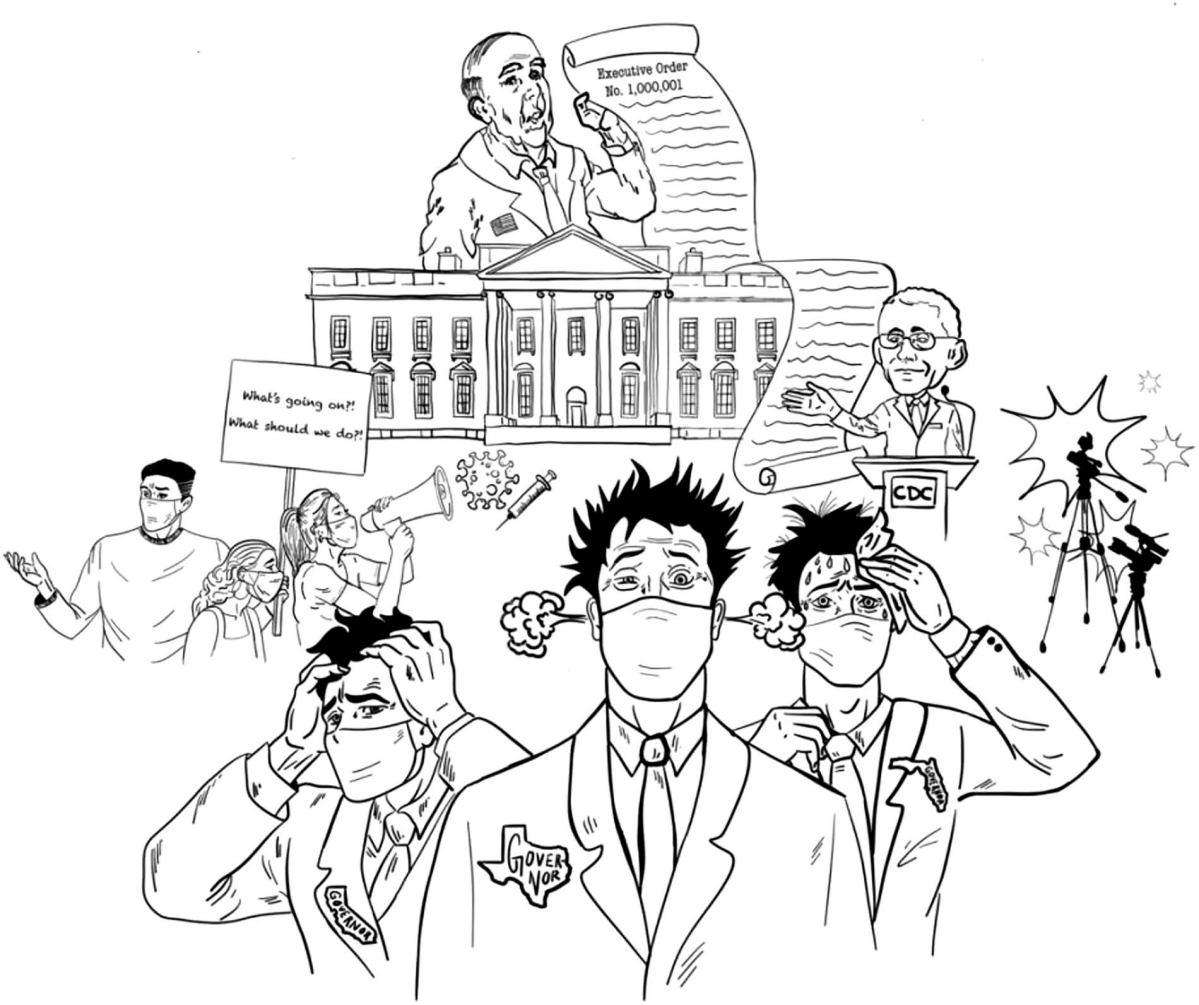
The key motivation for this work is that COVID-19 vaccine acquisition, deployment, and uptake in the United States were largely managed by state and local governments, which had to navigate between federal policies and local needs. Each state developed and implemented its own strategy to inform and support its residents. This dataset preserves contemporaneous documents from state governors and state health departments, capturing their efforts around COVID-19 vaccine communication and rollout^12^. It is designed to support research on improving communication between governments and citizens during public health crises, including questions about how socioeconomic and geographic factors shape messaging and outreach, how people perceive and trust government institutions and leaders, and systematic patterns of government action. Original illustration by Avalon S. Moore.

We present a structured dataset of 5,223 public documents from the initial stages of the COVID-19 Vaccination Program, published online by the U.S. state governors’ offices and respective health departments. At present, this dataset is a completed snapshot of that defined time period. We do not plan to add additional documents or to extend the temporal coverage, but we provide full documentation so that others may build upon or update the dataset in future work. This open-access resource, available through the Qualitative Data Repository (QDR), supports research aimed at improving communication between state governments and their citizens during times of public health crises, fostering greater public trust in and efficacy of health initiatives, as illustrated in Fig. 1.

## Methods

### Temporal coverage of the database

We focused on publications from state governors and state departments of health published online between December 2020, when the first COVID-19 vaccines were granted EUA, and September 2021, when multiple vaccines were broadly available and often mandated. Thus, the temporal span of our study covered the period of most active efforts made to implement the vaccination program by states. This period included three key phases^1^:

- Phase I (December 2020 – Spring 2021): Vaccine Approval and Initial Rollout
- Phase II (Spring 2021 – mid-Summer 2021): Broadening Access
- Phase III (mid-Summer 2021 – early Fall 2021): Reaching Vaccine-Hesitant Individuals and Groups with Limited Access

### Geographical coverage of the database

The initial wave of data was collected to supplement a longitudinal quantitative dataset from a prospective study that examined the evolution of perceptions regarding vaccine safety and the decision to get vaccinated in 318 individuals from 46 states^13^. It covered most of the U.S. states, excluding Alaska, Idaho, North Dakota, and Vermont, and including the District of Columbia. Data was collected between September 2021 and July 2022. Subsequently, the dataset was expanded into a comprehensive, standalone collection that encompassed all 50 U.S. states and the District of Columbia. Wave 2 covered the remaining 4 states and was collected between September 2023 and July 2024. Because some publications may have been removed from the official websites of the state governors and the states departments of health over time, a possible limitation is that the coverage in Wave 2 might be less complete than in Wave 1.

### Data mining protocol

We intentionally relied on standard Google Search to identify documents, as this approach reflects how members of the general public are most likely to seek official information about COVID-19 vaccination programs.

Within each wave, data collection was conducted in four steps: initial search, selection, classification, and quality control. To ensure that uniform criteria were applied to all states, a general data mining protocol was developed (Supplemental Information S1). Figures S1–S6 (located within Supplemental Information S1) depicts the standardized protocol for categorization of state publications, and Figure S7 depicts the overall workflow.

First, during the initial search, data were collected by the first author (AM) from the respective state’s governor’s and department of health websites, using Google Search with the ‘Custom range’ tool, in week-long increments (e.g., 12/01/2020-12/07/2020), and key word <vaccine>. For each unique source/week search combination, we manually inspected all documents up to the final available page in Google Search, systematically collecting publications referring to the COVID-19 vaccine.

Second, during the selection step, search results were reviewed to satisfy the pre-specified inclusion/exclusion criteria by different authors in charge of different states (by AM, BV, FFB, PW, LVDM, and AG). We included: (i) publications from state-run websites ending in ‘.gov’ that relate to the COVID-19 vaccination program; (ii) for New Mexico, publications from the department of health website, which ends in ‘.org’; (iii) for Minnesota, publications from the department of health website, which ends in ‘.us’. We excluded: (i) publications from individual counties, universities, and other organizations not related to state government branches or health sectors, (ii) videos with no transcription posted by state government offices or the state departments of health, (iii) publications with no text, (iv) publications referring to vaccines other than the COVID-19 vaccine (e.g., rabies vaccines, influenza vaccines), (v) publications not in the English language. The included publications are organized by state → month → week of the publication.

Third, during the classification step, all publications were categorized into six groups based on publication type and source (by AM, BV, FFB, PW, LVDM, and AG): (1) communications from the governor and other state officials, (2) communications from the state department of health or other state health authorities, (3) policies issued by the governor or other state officials, (4) policies issued by the state department of health or other state health authorities, (5) flyer (1–3 pages, primarily visual content), and (6) milestone (publications presenting quantitative data in tables or graphs). The criteria for classification are outlined in Table 1. We recognize that in some instances, the application of these criteria involved a degree of subjectivity. To mitigate this subjectivity, all coders followed a unified classification protocol, and we relied on manual review—which remains the gold standard for policy-document classification because it preserves contextual meaning and interpretive accuracy that automated or AI-based tools can miss^14,15^.

**Table 1:** Inclusion and exclusion criteria were used to classify documents into six categories.

Category	Include	Exclude
Information from the governor and other state officials (Info Gov)	Press releases or documents released by state governments, state government officials, or any state sector not associated with the department of health.	Press releases from the department of health or any branch connected to that department.
Information from the state department of health or other state health officials (Info Health)	Press releases or documents released by state departments of health or departments of public health (including departments of mental health or any sect of the state’s public health division).	Press releases from any government branch not associated with the department of health.
Policy from the governor or other state officials (Policy Gov)	Directives (e.g., executive orders, memorandums, bills, etc.) issued by a governor or state legislature that regulates operations of the state government.	Orders, memorandums, bills that are released specifically from the department of health.
Policy from the state department of health or other state health officials (Policy Health)	Directives issued by the state’s department of health that regulates operations, the state’s health sectors (e.g., hospital workers, pharmacists, etc.), and aspects of citizen life.	Orders, memorandums, or bills that are released specifically from the state government, even if they mention the department of health.
Flyer	Advertising FAQs and information on vaccination (Often utilizing graphic images; e.g., clip art) and listed in bullet points, not always in full-length sentences or paragraphs, commonly 1–3 pages maximum).	Documents that only consists of accessible/active links or QR codes, and do not include any other information.
Milestone	Documents that present numerical, statistical, or raw data without substantial narrative text (e.g., tables, weekly vaccination dashboards, cumulative counts, charts, or brief data summaries).	Documents that have more textual paragraphs than statistical representations (i.e., graphs and tables).

Each publication was saved in a Portable Document Format (.pdf). Each file name preserved the original title of the document with added prefix “Moore-et-al_<2 letter state abbreviation>_< mm><yy>.” For instance, if a document title was “Alabama Medicaid” and it was published in Alabama in December of 2020, then the name of the file was “Moore-et-al_AL_1220_Alabama Medicaid.” If several documents were published in the same month with the same title, a numerical suffix was added to indicate the order of the publication. For instance, a document “Moore-et-al_CT_0321_Governor Lamont Provides Update on Connecticut’s Coronavirus Response Efforts_01” was released by Governor Lamont before the document “Moore-et-al_CT_0321_Governor Lamont Provides Update on Connecticut’s Coronavirus Response Efforts_02.” The prefix with the first author’s last name was added as a part of QDR’s naming convention in order to show provenance when the data are downloaded by secondary users.

### Quality control

Quality control was done in three stages. First, the first author (AM) met weekly with other contributing authors (BV, FFB, PW, LVDM, and AG) in charge of different states during the selection and classification phases to review their progress and clarify the inclusion/exclusion and classification criteria. Second, the first and the last author (HP) met quarterly, to review the selected and classified publications from randomly selected states, to resolve any ambiguities, and to make the final decisions surrounding selection and classification of the publications.

Third, the final review was done prior to depositing data to the repository by three co-authors (AM, AG, and HP). The focus was on ensuring that all collected records were sorted appropriately. To that end, the last author generated a list of all files in the master folder and checked if each unique publication name appeared exactly twice on the list: once in the raw folder and once in one of the sorted folders. 797 documents did not satisfy these criteria (~15%). The two co-authors (AM and AG) reviewed these documents, identified 112 problems with inconsistent naming of files (~ 2%), and discussed how the remaining 685 publications (~13%) should be sorted. In the case of disagreements, the last author served as a tie breaker.

Fourth, we anticipated that over time some COVID-19 vaccination–related documents would be removed from official state websites as outdated or as a part of common broader website reorganizations. To assess the persistence of online availability, we conducted a selective follow-up search in December 2025. This analysis was not intended to reconstruct the full universe of deleted documents, but rather to evaluate whether previously collected documents remained findable using the same search strategy.

Specifically, we randomly selected two states with larger populations (Connecticut and Maryland) and two states with smaller populations (Montana and Wyoming) from those assessed in 2021, as well as all four states assessed in 2024 (Alaska, Idaho, North Dakota, and Vermont). For each state, we searched for documents issued by the governor’s office and the state department of health during three reference periods corresponding to key phases of the vaccination program: December 2020 (Phase I), May 2021 (Phase II), and September 2021 (Phase III), using the same Google Search approach as in the original data collection.

**Fig. 2:**
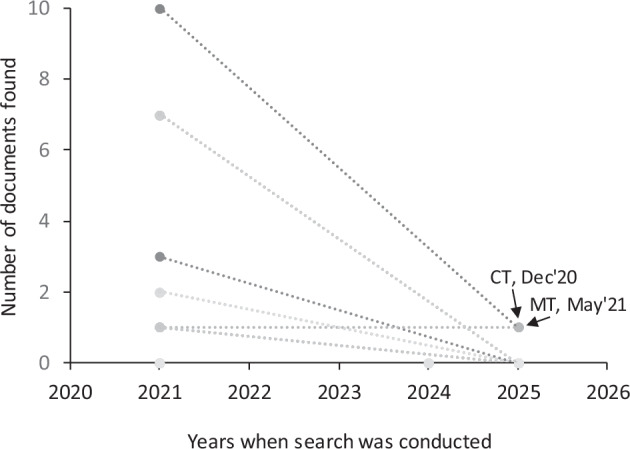
Temporal patterns in the online availability of documents from the dataset^12^. The test set included four states assessed in 2021 (Connecticut, Maryland, Montana, and Wyoming) and four states assessed in 2024 (Alaska, Idaho, North Dakota, and Vermont). The observed decline in document availability over time is consistent with expectations that COVID-19 vaccination–related communications are removed from official state websites as they become outdated.

Across these states and periods, 43 documents were identified during the original 2021/2024 searches. When repeating the search in December 2025, only two of these documents remained available online: one flyer from Montana and one press release from the governor’s office in Connecticut (Fig. 2). This pattern suggests substantial attrition of online availability over time, with no clear qualitative differences between the selected rural and urban states in this limited assessment.

In addition, the 2025 search identified four documents that had not been captured previously: two from Connecticut (one press release from the state department of health and one policy from the governor’s office) and two from Maryland (one press release from the state department of health and one press release from the governor’s office). These newly identified documents were added to the dataset. Importantly, this follow-up analysis does not allow inference about systematic deletion patterns across states or population characteristics but instead highlights the dynamic nature of online government communication and the importance of time-specific data capture.

## Data Records

### Database availability

The database is freely available through the Qualitative Data Repository (QDR) Main Collection. It contains 5,223 unique documents published by state governments and state departments of health across all 50 U.S. states and the District of Columbia between December 2020 and September 2021. All documents in this dataset were obtained from publicly accessible U.S. state government websites and are public records intended for unrestricted public dissemination^16^. Accordingly, all materials included in the dataset are legally sharable and redistributable under the terms of the CC-BY license. No subscription is required to access the database.

### Folder structure

Files are organized first by state and then by month (e.g., AK → 2021_01). Within each monthly directory, two types of subfolders are provided:

1. *raw/* – Contains the original, unclassified documents organized by week (e.g., *Week_2/*).
2. Classified folders – Documents assigned to one of six categories:

- *Info/gov -* Press release from Governor’s Office
- *Info/health -* Press release from State Health Department
- *Policy/gov* - Policy from Governor’s Office
- *Policy/health -* Policy from State Health Department
- *Milestone*
- *Flyer*

Each document is therefore stored twice within the dataset:

1. an unprocessed version in the *raw/* folder, and
2. a categorized version in the appropriate classification folder.

This structure enables users to review the original files and apply their own classification schemes if desired.

### Accompanying metadata

The data project also includes the following documentation files: a full inventory of the items in both Excel and CSV formats, containing a full list of publications with their upload dates, as well as the number of publications by state and by type, organized by month from December 2020 to September 2021; the Classification Protocol used by the team; a Data Narrative describing briefly the data collection methodology; an administrative README file which contains a full directory map of the data files.

## Data Overview

### Description of the database

**Fig. 3:**
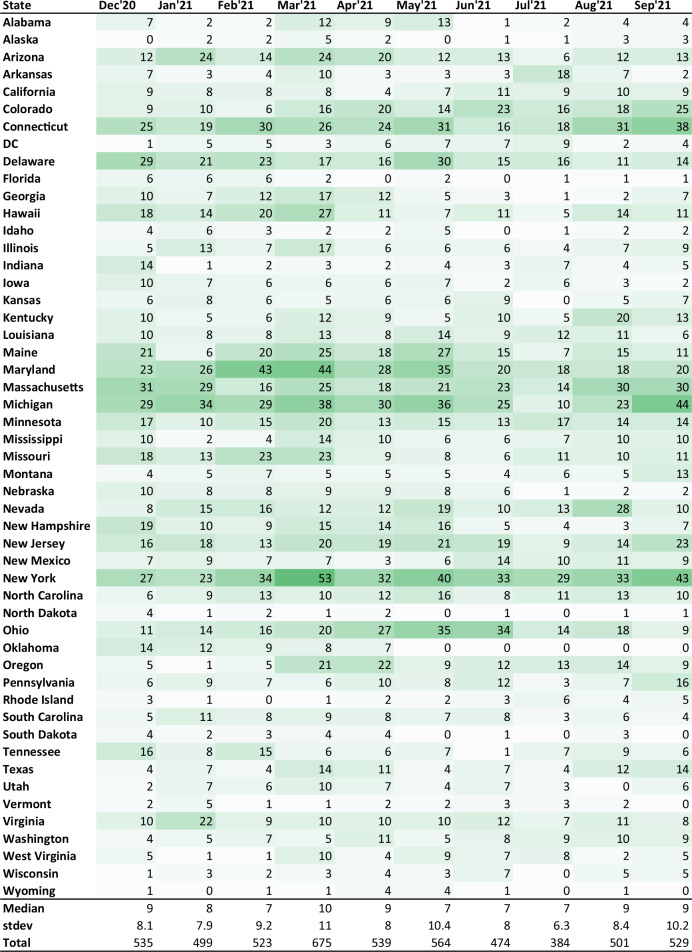
The distribution of the final 5,223 publications across states, and months. Cells are color-coded by value, with lighter shades representing lower numbers and darker shades indicating higher numbers.

The distribution of all 5,223 documents across states and months is shown in Fig. 3. Figure 3 illustrates that the database has an uneven temporal and geographic coverage, from dozens (New York in March 2021: 53; Maryland in March 2021: 44; Michigan in September 2021: 44; Maryland in February 2021: 43) to a handful of publications, or none (Alaska in December: 0; Wisconsin in July 2021: 0; Florida in April 2021: 0; Idaho in June 2021: 0). The largest number of publications was issued in March 2021, during the transition from Phase I to Phase II, when states were preparing for and entering the period of broadening vaccine access to additional population groups (ranging from 1 in North Dakota, Rhode Island, Vermont, and Wyoming to 53 in New York; median = 10). The smallest number of publications were issued in July 2021 (from 0 in Kansas, North Dakota, Oklahoma, South Dakota, Wisconsin, and Wyoming to 29 in New York; median = 7), which corresponds to early Phase III, when efforts shifted toward reaching vaccine-hesitant groups before pediatric vaccines became available. Note that the initial search was conducted in Google, but we additionally searched the websites of the state governors and departments of health from the respective states. Thus, our search was done more carefully than would be done by a typical news consumer and is more likely to yield more accurate results. Still, we caution against the interpretation of no relevant publications found by our team to mean no relevant publication was published online. Figure 3. The distribution of the final 5,223 publications across states, and months. Cells are color-coded by value, with lighter shades representing lower numbers and darker shades indicating higher numbers.

**Fig. 4:**
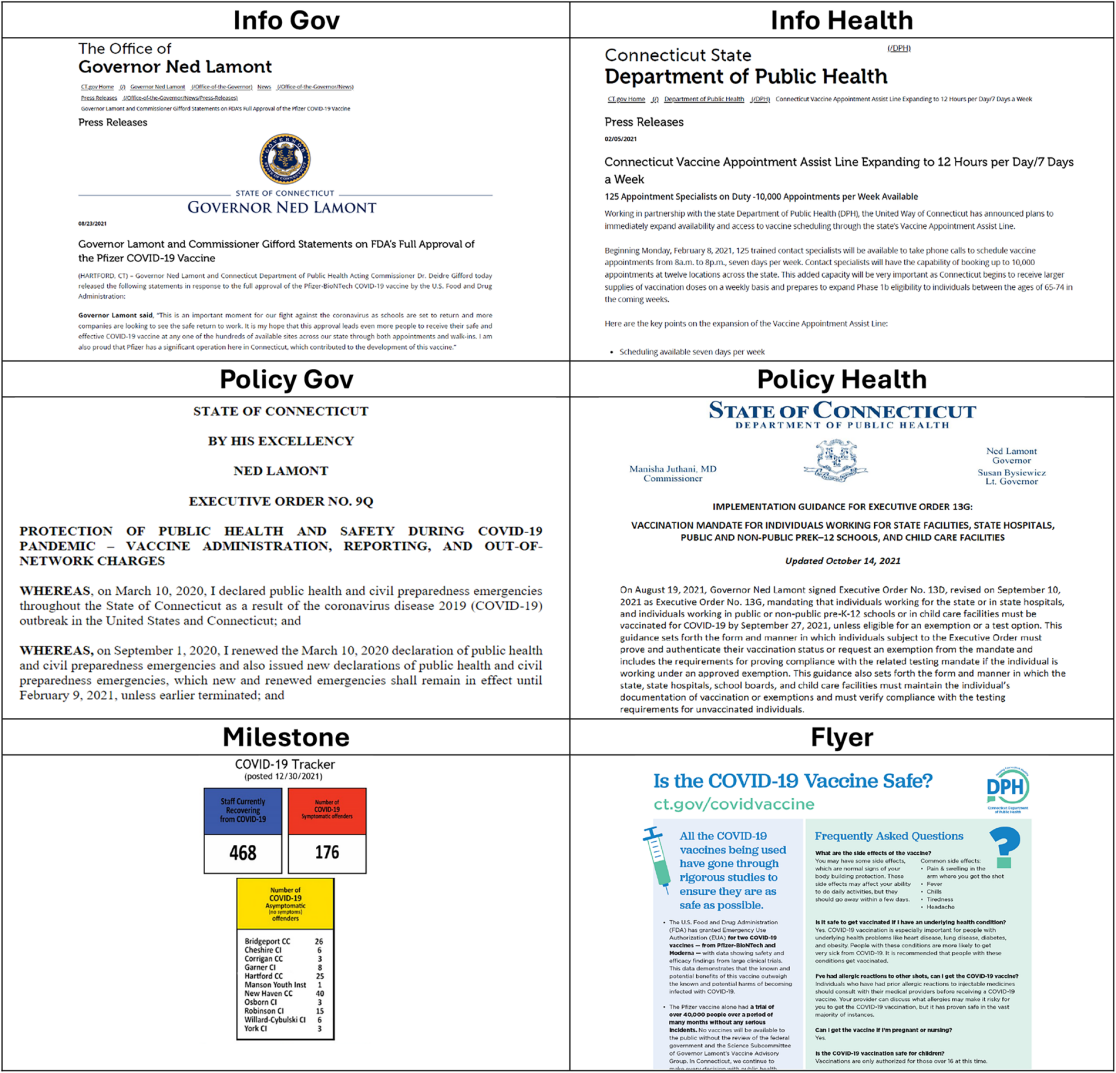
Representative examples of publications from each of six categories.

Additionally, we classified all documents into categories – policy from governor's office, policy from state health departments, press releases from governor's office, press releases from state health departments, milestone, and flyer—as detailed in Methods. The primary reason for classifying the publications into these six categories was to create groups of documents that use more homogeneous linguistic styles. For instance, press releases and informational documents tend to rely on more accessible language while policy documents tend to use more formal and prescriptive language. This was done to enable future analyses of linguistic patterns. Figure 4 illustrates the differences across these six categories, using data from the state of Connecticut.

### Limitations in using the database

Users of the database must be aware of the following limitations:

1. Coverage: The database includes written materials posted on official state government websites (e.g., governor’s office, state department of health). Televised briefings, radio segments, and in-person addresses are not included if transcripts were not available on these websites.
2. Search dependence: Documents were identified through Google Search between September 2021 and July 2024. All Google searches were conducted from New Haven, Connecticut. Because indexing varies by location^13^ and overtime, absolute counts may not be strictly comparable across states. Although we cannot access or control the proprietary algorithms that drive these variations, all searches were conducted from the same geographic location and using consistent devices, browser settings, and search terms. As a result, within-state proportions (e.g., governor vs. department of health releases) are more reliable than cross-state comparisons. This limitation is consistent with prior reports documenting geographic variation in Google Search results^17^.
3. Retention considerations: Some states may have removed or overwritten older postings as no longer relevant. Our data collection began in September 2021; while temporal proximity likely allowed to preserve most of the online publications, some documents could have been already removed. The probability of removal increases with time; thus, this caution especially applies to data from Alaska, Idaho, North Dakota, and Vermont, since these data were collected in 2024. Thus, absolute counts may not be comparable across states; within-state proportions (e.g., governor vs. department of health releases) are likely to be more reliable.
4. Format and language: Video briefings without posted transcripts could not be archived or searched in a consistent way and are not included. When non-English materials appeared alongside an English version, we retained the English posting to avoid double-counting; non-English-only materials were not included.
5. Source domains: We restricted collection to documents hosted on official state government domains. We did not systematically search non-governmental sites (e.g., hospital systems, community partners, document-sharing services, or social media platforms), so web-only flyers and other materials distributed solely through those channels fall outside the scope of the dataset.

## Technical Validation

### Data accuracy and curation

To increase the accuracy and internal consistency of our database, and because of a certain degree of subjectivity in categorization of the documents into six groups, the last author re-assessed approximately 23% of the finalized dataset (N = 1,187)^18^. The re-assessment was conducted blindly: the re-assessor classified each document without seeing its previously assigned category. Only after this independent classification was completed was the reassessment compared to the original coding. The column “Re-Assessor” in the files that list of all documents indicates whether this re-assessment was performed for a given article (initials “HP” indicated who conducted the reassessment). The re-assessor examined each of the selected documents following the study data mining protocol (see Supplemental Information S1) and compared their assessment with the already assigned classification (reported in the column “Agreement”). In the case of disagreement, the re-assessor suggested the alternative categorization in the column “Alternative category.”

The agreement rate between the initial assessment and re-assessment was 96% [95% CI, 95%–97%], indicating an estimated 4% subjectivity in the categorization process. The confidence intervals were calculated based on the variance estimate.

Additionally, we evaluated whether general patterns in our dataset are consistent with theoretical expectations or patterns reported by prior studies that relied on other data sources (i.e., triangulation of evidence). Specifically, we focused on regional differences in responses of state governors and state departments of health to challenges of the COVID vaccination roll out.

Much prior literature in Sociology and Public Health has documented that during crises, individuals in urban contexts are more likely to rely on formal institutions than those in rural settings^19,20^. Likewise, we would expect formal institutions, such as state governors, to engage more actively in communication with the general public in urban contexts compared to rural ones. The lack of targeted communication campaigns from the governors’ office could have contributed to slower vaccine uptake in rural areas^21^. We do not anticipate seeing the same drastic difference in communication from the state departments of health, since during the COVID-19 vaccination program they were proactively engaged in interactions with their federal counterpart and were continuously receiving instructions on what information has to be shared and when with general public^22^.

To test whether our dataset aligns with these theoretical expectations, we incorporated data on the Index of Relative Rurality (IRR)^23^. The IRR is a continuous, threshold-free, and unit-free measure of rurality calculated at the county level. The IRR dataset is openly available through the Purdue University Research Repository (PURR)^23^. First, we computed the average IRR for each state; based on this state-level IRR, we classified 50 states and the District of Columbia into three groups of 17: more urban, mixed, and more rural. These indices are included in the dataset available on GitHub (see below).

**Fig. 5:**
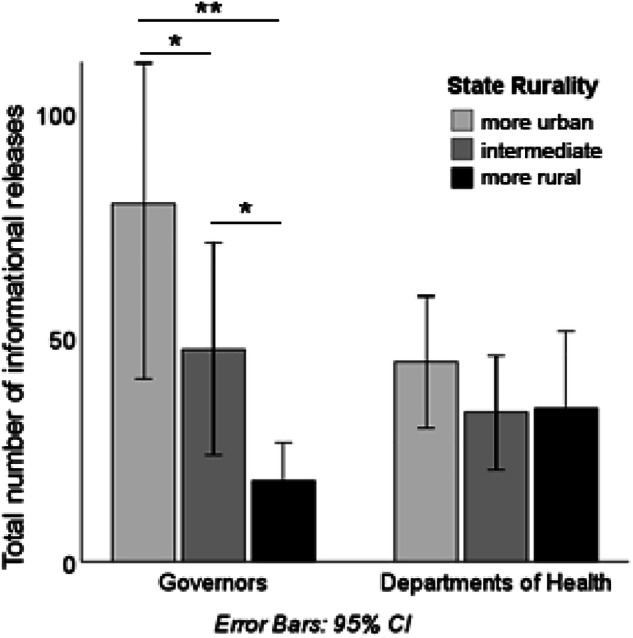
Regional differences – more rural versus more urban states – in the number of publications released by state governors and state departments of health that were categorized as “Information.” Bars depict estimated marginal means from a repeated measures ANOVA; with the number of publications as a dependent variable, the source of information (governors/ state departments of health) as repeated measures, and the rurality level as a between factor. Note. Significance levels: **p < 0.01, *p < 0.10.

A repeated measures ANOVA, conducted in SPSS 29.0, revealed a monotonic pattern consistent with our expectations (see Fig. 5). Governors from more urban states released significantly more informational documents than those from more rural states (Cohen’s d = 1.2, p < 0.001). Governors from the mixed group of states released fewer documents than those from more urban states (Cohen’s d = 0.62, p = 0.07) but more than those from more rural states (Cohen’s d = 0.57, p = 0.10). In contrast, and as expected, the number of publications from state departments of health did not differ significantly across the three groups, although departments of health in more urban states released slightly more publications than their counterparts in more rural states (Cohen’s d = 0.29, p = 0.26).

## Supplementary information

Supplementary Information S1

## Data Availability

The data described here can be found and accessed freely at Moore, Avalon S.; Bridget, Vitu; Fraizer-Bisner, Felicia; Williams, Peter J.; van der Merwe, Lucy; Gouda, Abdelrhman; Pittenger, Christopher; Pushkarskaya, Helen. 2025. “Data for: COVID Diaries, State Response to COVID Vaccination Program, December 2020 to September 2021”. Qualitative Data Repository. 10.5064/F6G2PETF. QDR Main Collection. V3. There is a related dataset with a different type of data (inventory of media responses to the COVID-19 pandemic during the same period). Since those data are not the focus of this article, we do not refer to the dataset within the text.

## Code availability

The data file to generate Fig. 4 is available in GitHub: https://github.com/HNP-data/COVID-DIARIES.git. Analyses were conducted in IBM SPSS 26.0.0. No custom code was generated for this work.
