# Supplementary material for: COVID Diaries, State Response to COVID Vaccination Program, December 2020 to September 2021
Source: Sci Data. 2026 Mar 20;13:775. doi: 10.1038/s41597-026-06975-0 (PMC13199395; doi:10.1038/s41597-026-06975-0)

Supplementary Information for

COVID Diaries, State Response to COVID Vaccination Program, December 2020 to September 2021

Moore, et al.

Supplementary materials include:

1) **Supplementary text.** A “**STANDARDIZED PROTOCOL FOR CATEGORIZATION OF STATE PUBLICATIONS**”, listing the protocol procedure for categorizing U.S. state publications.
2) **Figure S1**
3) **Figure S2**
4) **Figure S3**
5) **Figure S4**
6) **Figure S5**
7) **Figure S6**
8) **Figure S7**

1 – STANDARDIZED PROTOCOL FOR STATE PUBLICATION ORGANIZATION

a. **State selection**
Begin by navigating to the parent directory that contains folders for all U.S. states. Locate and select the folder corresponding to your assigned state. All subsequent organization steps will take place within this state-specific folder.

b. **Chronological starting point**
Within your assigned state folder, identify the folder labeled December 2020. December 2020 is the first chronological month included for each state and should always serve as the starting point for the organization process.

c. **Folder creation and standardized structure**
Once you open the December 2020 folder for your assigned state, you will see a folder labeled "Raw," which contains all files that will be organized into the standardized folder structure. Before opening the Raw folder, first create the following set of subfolders within the December 2020 directory. This same folder-creation process should be repeated for each subsequent month.

- Create four primary folders:
    - Flyer
    - Milestone
    - Info
        - Within the Info folder, create two subfolders:
            - Info_Gov
            - Info_Health
    - Policy
        - Within the Policy folder, create two subfolders:
            - Policy_Gov
            - Policy_Health

This results in a total of six destination folders for sorted publications:

- Flyer
- Milestone
- Info_Gov
- Info_Health
- Policy_Gov
- Policy_Health

d. **Raw folder overview**
After creating the standardized folder structure, navigate to the raw folder within the December 2020 directory. Inside the raw folder, you will find four subfolders labeled:

- Week 1
- Week 2
- Week 3
- Week 4

Note: Each weekly folder contains unsorted publications that must be reviewed and categorized.

e. **Weekly sorting procedure**
Begin with Week 1 and repeat the following steps for each week (Weeks 1-4). Once inside a weekly folder, select the first file listed. The same evaluation and sorting criteria should be applied to every file within that week before moving on to the next week.

f. **File classification and sorting criteria**
For each selected file, follow this decision-making process:

g. **Determine the publication's visual format**:

- Step 1. Assess content type for Flyer or Milestone shown in Figure S1 and S2.
    - If not Flyer or Milestone, disregard Step 2 and decide whether the document refers to Info or Policy (refer to Figures S3 and S4 before moving to Step 3).
- Step 2. If the file meets criteria for Flyer or Milestone, *copy* it directly into the corresponding folder without *moving* it from its original location within the Raw folder.

h. **Determine publishing authority**

- Step 3: Assess whether the document derived from the state's government offices or the department of health.
    - If the publishing source is not explicitly stated within the title, body, or visible links, refer to the content of the document.
- Files discussing government actions, executive decisions, or legislative processes should be categorized as Gov, see Figure S5.
- Files emphasizing health guidance, disease prevention, vaccination procedures, or public health measures should be categorized as Health, see Figure S6.

i. **File placement**
Once the publication type and source have been determined, copy (do not move) the file into the appropriate destination folder among the six created earlier.

j. **Final folder cleanup**
After all files from Weeks 1-4 have been reviewed and copied into their appropriate folders, return to the December 2020 directory. Review the six destination folders and delete any folders that remain empty to ensure a clean and finalized directory structure.

k. **Data-flow Protocol**
The data-flow process followed a staged, multi-wave workflow beginning with two sequential waves of data collection and categorization. Wave 1 data collection was completed by AM between September 2021 and July 2022 for states ranging from Alabama to Massachusetts, followed by Wave 1 data categorization completed by BV between September 2022 and March 2023. In parallel, Wave 2 data collection was completed by AM between September 2023 and July 2024 for states ranging from Michigan to Wyoming, with Wave 2 data categorization completed by FFB and LVDM between September 2023 and July 2024. Outputs from both waves were integrated and subjected to centralized quality control conducted by AM, AG, and HP between July 2024 and September 2024 across all states. Finally, a targeted re-assessment phase was completed by HP between September and October 2024 for a subset of states (AL, AK, AZ, AR, CA, KS, KY, LA, ME, NM, NC, ND, and OH) to verify coding accuracy and ensure analytic readiness. For a visual depiction of this protocol, see Figure S7.

2 - Figure S1

Flyer: Typically, 1–3 pages in length; contains visual elements such as graphics, charts, icons, bullet points, or simplified messaging.

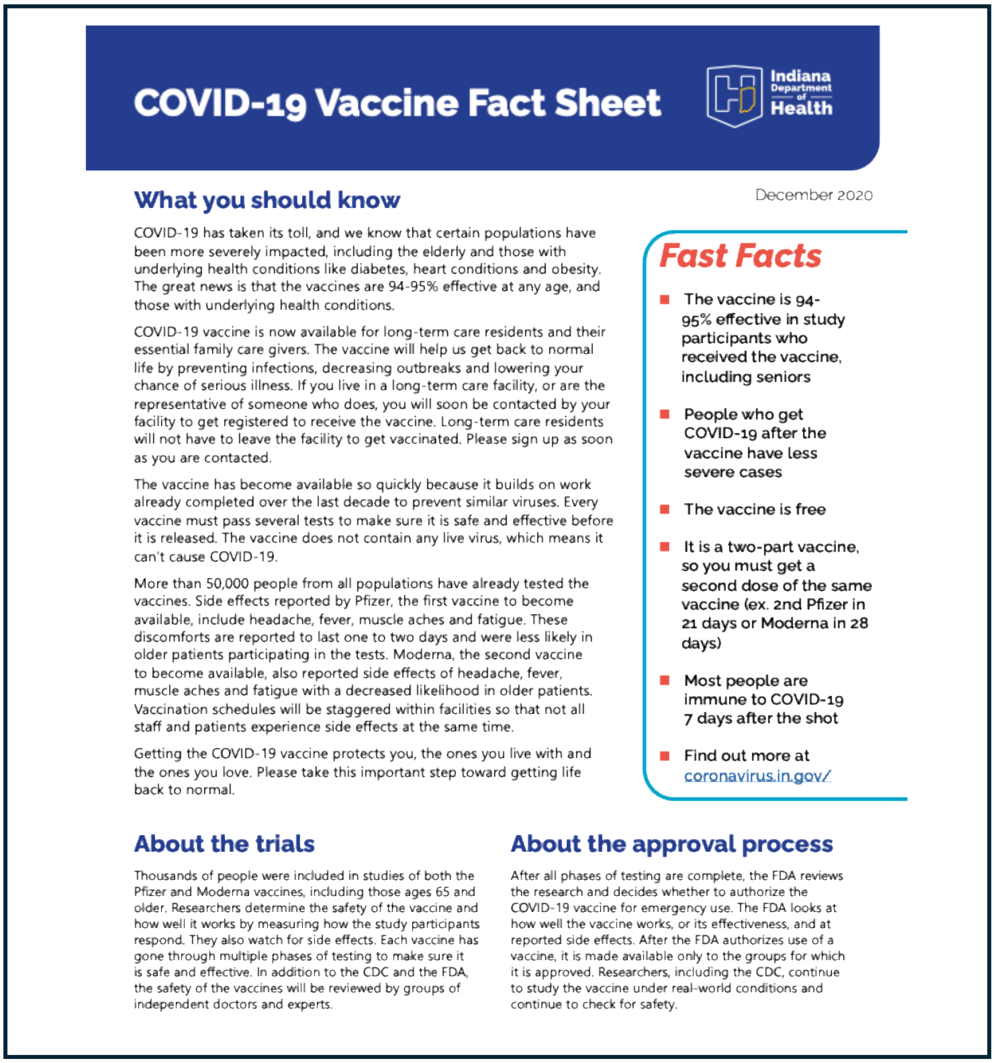

COVID-19 Vaccine Fact Sheet

Indiana Department of Health

December 2020

What you should know

COVID-19 has taken its toll, and we know that certain populations have been more severely impacted, including the elderly and those with underlying health conditions like diabetes, heart conditions and obesity. The great news is that the vaccines are 94-95% effective at any age, and those with underlying health conditions.

COVID-19 vaccine is now available for long-term care residents and their essential family care givers. The vaccine will help us get back to normal life by preventing infections, decreasing outbreaks and lowering your chance of serious illness. If you live in a long-term care facility, or are the representative of someone who does, you will soon be contacted by your facility to get registered to receive the vaccine. Long-term care residents will not have to leave the facility to get vaccinated. Please sign up as soon as you are contacted.

The vaccine has become available so quickly because it builds on work already completed over the last decade to prevent similar viruses. Every vaccine must pass several tests to make sure it is safe and effective before it is released. The vaccine does not contain any live virus, which means it can't cause COVID-19.

More than 50,000 people from all populations have already tested the vaccines. Side effects reported by Pfizer, the first vaccine to become available, include headache, fever, muscle aches and fatigue. These discomforts are reported to last one to two days and were less likely in older patients participating in the tests. Moderna, the second vaccine to become available, also reported side effects of headache, fever, muscle aches and fatigue with a decreased likelihood in older patients. Vaccination schedules will be staggered within facilities so that not all staff and patients experience side effects at the same time.

Getting the COVID-19 vaccine protects you, the ones you live with and the ones you love. Please take this important step toward getting life back to normal.

Fast Facts

- The vaccine is 94-95% effective in study participants who received the vaccine, including seniors
- People who get COVID-19 after the vaccine have less severe cases
- The vaccine is free
- It is a two-part vaccine, so you must get a second dose of the same vaccine (ex. 2nd Pfizer in 21 days or Moderna in 28 days)
- Most people are immune to COVID-19 7 days after the shot
- Find out more at coronavirus.in.gov/

About the trials

Thousands of people were included in studies of both the Pfizer and Moderna vaccines, including those ages 65 and older. Researchers determine the safety of the vaccine and how well it works by measuring how the study participants respond. They also watch for side effects. Each vaccine has gone through multiple phases of testing to make sure it is safe and effective. In addition to the CDC and the FDA, the safety of the vaccines will be reviewed by groups of independent doctors and experts.

About the approval process

After all phases of testing are complete, the FDA reviews the research and decides whether to authorize the COVID-19 vaccine for emergency use. The FDA looks at how well the vaccine works, or its effectiveness, and at reported side effects. After the FDA authorizes use of a vaccine, it is made available only to the groups for which it is approved. Researchers, including the CDC, continue to study the vaccine under real-world conditions and continue to check for safety.

3 - Figure S2

Milestone: Contains raw numerical data (e.g., case counts, vaccination totals, percentages) with minimal to no narrative text.

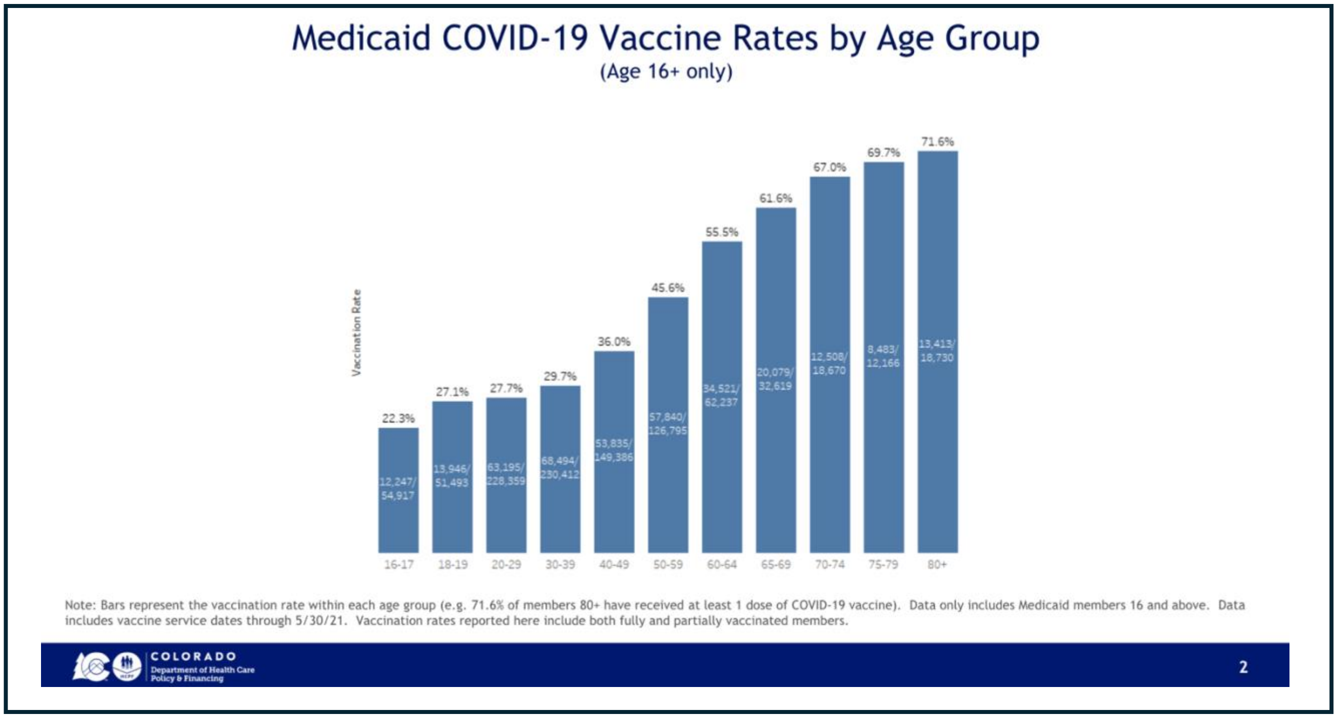

4 - Figure S3

Info: Provides informational or educational content (e.g., vaccine explanations, eligibility information, safety guidance).

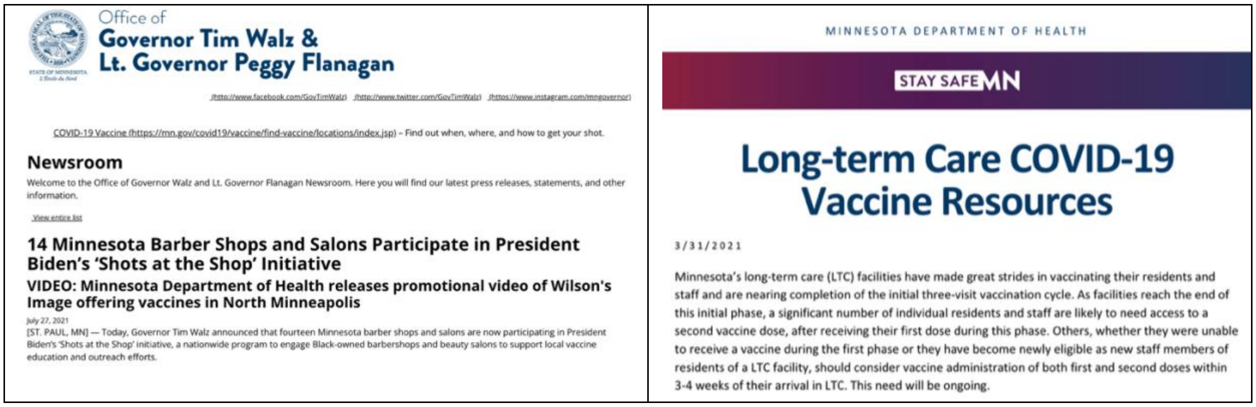

5 - Figure S4

Policy: Outlines formal guidance, regulations, mandates, or procedural rules related to civilian life, institutions, or public behavior.

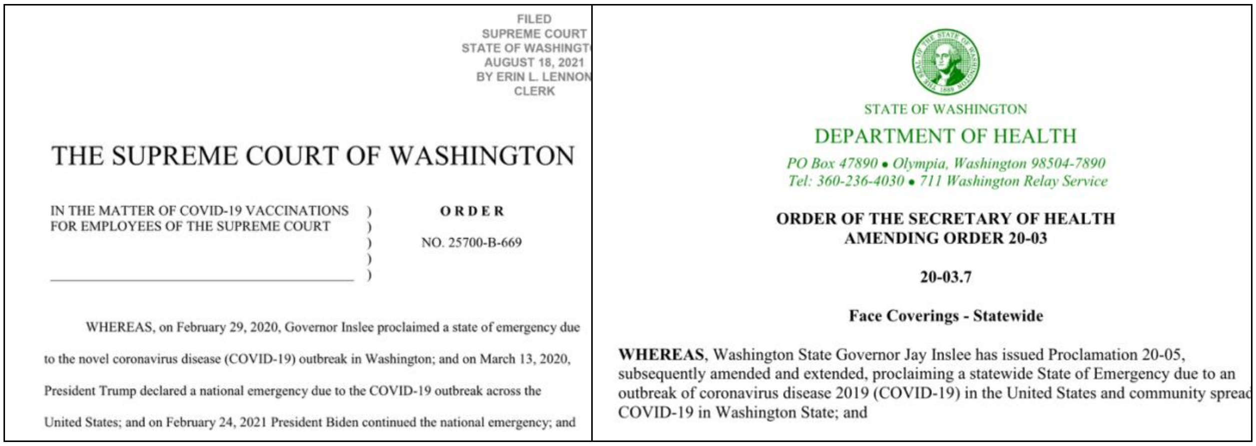

FILED
SUPREME COURT
STATE OF WASHINGT
AUGUST 18, 2021
BY ERIN L. LENNON
CLERK

THE SUPREME COURT OF WASHINGTON

IN THE MATTER OF COVID-19 VACCINATIONS FOR EMPLOYEES OF THE SUPREME COURT

ORDER

NO. 25700-B-669

WHEREAS, on February 29, 2020, Governor Inslee proclaimed a state of emergency due to the novel coronavirus disease (COVID-19) outbreak in Washington; and on March 13, 2020, President Trump declared a national emergency due to the COVID-19 outbreak across the United States; and on February 24, 2021 President Biden continued the national emergency; and

STATE OF WASHINGTON
DEPARTMENT OF HEALTH
PO Box 47890 • Olympia, Washington 98504-7890
Tel: 360-236-4030 • 711 Washington Relay Service

ORDER OF THE SECRETARY OF HEALTH
AMENDING ORDER 20-03

20-03.7

Face Coverings - Statewide

WHEREAS, Washington State Governor Jay Inslee has issued Proclamation 20-05, subsequently amended and extended, proclaiming a statewide State of Emergency due to an outbreak of coronavirus disease 2019 (COVID-19) in the United States and community spread COVID-19 in Washington State; and

6 - Figure S5

Gov: Issued directly by a state government entity (e.g., Governor's office, state legislature, executive orders).

FILED
SUPREME COURT
STATE OF WASHINGTON
AUGUST 18, 2021
BY ERIN L. LENNON
CLERK

THE SUPREME COURT OF WASHINGTON

IN THE MATTER OF COVID-19 VACCINATIONS FOR EMPLOYEES OF THE SUPREME COURT

O R D E R

NO. 25700-B-669

WHEREAS, on February 29, 2020, Governor Inslee proclaimed a state of emergency due to the novel coronavirus disease (COVID-19) outbreak in Washington; and on March 13, 2020, President Trump declared a national emergency due to the COVID-19 outbreak across the United States; and on February 24, 2021 President Biden continued the national emergency; and

7 - Figure S6

Health: Issued by a state department of health or public health authority.

MINNESOTA DEPARTMENT OF HEALTH

STAY SAFE MN

Long-term Care COVID-19 Vaccine Resources

3/31/2021

Minnesota's long-term care (LTC) facilities have made great strides in vaccinating their residents and staff and are nearing completion of the initial three-visit vaccination cycle. As facilities reach the end of this initial phase, a significant number of individual residents and staff are likely to need access to a second vaccine dose, after receiving their first dose during this phase. Others, whether they were unable to receive a vaccine during the first phase or they have become newly eligible as new staff members of residents of a LTC facility, should consider vaccine administration of both first and second doses within 3-4 weeks of their arrival in LTC. This need will be ongoing.

8 – Figure S7

Data-flow Process: Workflow of data collection, categorization, quality control, and re-assessment.

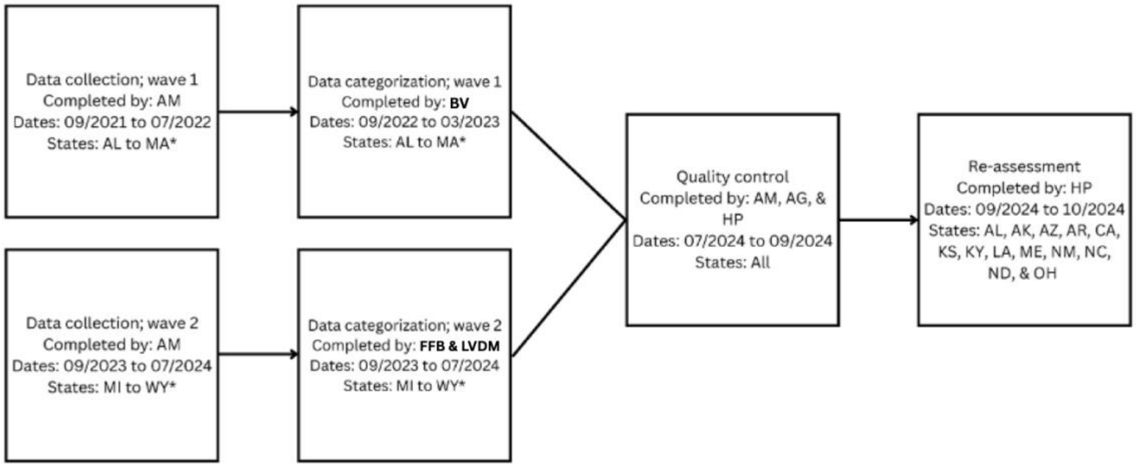

Supplement: Supplementary file 1 — Supplementary Information S1 [file 41597_2026_6975_MOESM1_ESM.pdf]
